# A clustering-based co-allocation of battery swapping stations and wind-photovoltaic plants in radial distribution systems

**DOI:** 10.1038/s41598-025-05440-z

**Published:** 2025-07-02

**Authors:** Hamed Shams, Naghi Rostami, Behnam Mohammadi Ivatloo

**Affiliations:** 1 Faculty of Electrical and Computer Engineering, University of Tabriz, Tabriz, 51666-16471 Iran; 2 School of Energy Systems, LUT University, Tabriz, Finland

**Keywords:** Electrical and electronic engineering, Power distribution

## Abstract

The growth of renewable sources and electric vehicles’ (EVs) load demand and associated uncertainties can stress the reliable network performance, such as uncertainty in both production and load sides, and power loss augmentation. These challenges can be mitigated by optimal planning considering variable output from wind and photovoltaic systems to meet the additional demand caused by EV charging. Swapping stations present an alternative solution for charging EVs that can lead to a different EV charging ecosystem. This study employs a stochastic clustering-based approach to optimally coallocate swapping stations, and wind-photovoltaic systems in networks. A K-means clustering method is implemented to classify price, energy demand, wind, and photovoltaic generation into appropriate clusters embedded into the particle swarm optimization (PSO) algorithm. The decision variables of PSO are the wind-photovoltaic system capacity and hybrid system placement to supply the EV load demand for battery swapping stations. The problem aims to maximize the net profit. The multi-criteria decision-making method, technique for order of preference by similarity to ideal solution, is applied to evaluate the results by considering all key influence criteria on the system’s performance. The performance of the proposed optimal co-allocation method on the IEEE 33-bus system has been investigated to demonstrate the effectiveness of integrating battery swapping stations into distribution systems.

## Introduction

The main motivations for distributed generation (DG) planning are loss reduction, reliability improvement, and voltage profile enhancement^1^. With increasing economic feasibility of wind farms, they have become an appealing alternative to conventional DG^2^. Beyond the geographical location of wind farms, determining their optimal size and placement within the power system is essential for ensuring both profitability and operational safety^3,4^. Additionally, the inherent variability of wind speeds significantly impacts the output power of wind farms and must be accounted for in the planning process^5^. Future power systems must consider a new source of uncertainty related to the load demand from plug-in electric vehicles (PEVs)^6^. The arrival and departure times, daily mileage, and vehicle types are heavily influenced by the behavioral patterns of PEV owners^7^. As a result, these parameters exhibit inherent uncertainty and should be modeled using probability distributions based on historical data to assess the uncertain load demand of PEVs accurately^8,9^. Since no international regulations mandate a specific charging pattern for PEVs, most owners are likely to charge their vehicles immediately upon returning home. This behavior often coincides with peak residential load hours, potentially straining the power grid during these times^10^. Consequently, PEVs substantially increase power consumption during peak hours, which are already associated with elevated demand levels. Optimal planning for the integration of embedded renewable generation within the distribution network is crucial for addressing this challenge. This approach helps minimize energy losses, reduce voltage fluctuations, and lower investment costs^11,12^. Optimal DG planning is a nonlinear, constrained, mixed-integer, and multi-objective problem, presenting significant challenges in finding a near-global optimal solution^13,14^. Therefore, employing an effective metaheuristic algorithm is essential for addressing this complexity. In the context of battery swapping stations, EV drivers can exchange their depleted batteries for fully charged ones at strategically located facilities, enhancing convenience and efficiency in battery management^15^. Swapping stations offer an alternative approach to charging EVs, potentially fostering the development of a distinct EV charging ecosystem. Swapping stations may be favored over traditional charging stations in scenarios where concerns arise regarding the upfront cost of EVs, charging speed, and intermittent electricity supply. Additionally, unlike conventional chargers, swapping stations enable faster battery replacement, providing fully charged batteries in a fraction of the time. For instance, electric taxis in urban public transport systems may favor swapping overcharging due to the time-sensitive nature of their operations^16^.

In a previous study, an operational model for a swapping station designed for a fleet of electric buses in public transportation was proposed^17^. The study aimed to optimize annual profits for the swapping station while minimizing the grid’s charging impact by examining the battery leasing model and the characteristics of electric city buses. Another scenario-based optimization algorithm was developed to allocate charging stations for a fleet of PEVs in a commercial area^18^. This approach focused on increasing the penetration of photovoltaic panels while reducing the negative effects of vehicular loads on the grid. A probabilistic load demand model was created using a multivariate stochastic method based on the Copula concept. To minimize energy loss and voltage deviation within the distribution system, the particle swarm optimization (PSO) algorithm was applied, and the model was validated through simulation results. In a related study, a model was developed using a mixed queuing network, incorporating an open queue for EVs and a closed queue for batteries^19^. Queueing network models were proposed as a framework for the design and modeling of swapping stations equipped with local charging capabilities. Experiments were carried out using a single swapping station, utilizing simulation techniques to offer valuable insights for the infrastructure planning of practical battery swapping services. In^20^, a real-time energy management strategy for a swapping station-based smart community microgrid (SCMG) was proposed. This strategy harnessed variable renewable energy sources to facilitate the charging of Electric Vehicle (EV) batteries while simultaneously supplying conventional residential loads. A novel Lyapunov optimization framework, grounded in queuing theory, was formulated to address the proposed model. This approach effectively streamlined complex energy scheduling by converting it into a single optimization problem, thereby ensuring its suitability for real-time applications. Simulation results demonstrated that using a swapping station for dual purposes significantly improved system economics and facilitated the integration of renewable energy, compared to isolated operations. In a research by Sarker et al.^20^ developed a mathematical model to manage uncertainty in swapping stations and optimize their operations. The model effectively managed fluctuating customer demand for fully charged batteries while optimizing the utilization of available batteries to reduce operational costs. This was achieved through demand-shifting strategies and energy sell-back mechanisms. The authors utilized mixed-integer linear programming (MILP) to address the scheduling problem for a single swapping station, integrating battery degradation into the model to achieve a practical and effective solution. Simulations demonstrated the model’s effectiveness and viability in minimizing operational costs.

While research on charging stations continues to grow, it is equally important to investigate other renewable energy sources, such as wind and solar photovoltaic (PV) power, to meet the energy demands of EV swapping stations. However, the inherent variability and intermittency of wind and solar PV energy pose significant challenges for power system operations, heightening the risks associated with operational decision-making.One key challenge is the accurate calculation of power flow. Traditional deterministic methods may no longer be suitable, as they cannot account for the uncertainties inherent in renewable energy sources^21,22^. Probabilistic techniques are essential for addressing load flow and optimal placement challenges under unpredictable conditions. A well-known method for reducing execution costs is data clustering. Previous studies have investigated the application of data clustering techniques to address various challenges, such as total transfer capability and power flow analysis. The data clustering technique efficiently manages large datasets, enabling the extraction of critical insights from complex information.

In this study, an effective methodology is proposed for the simultaneous co-allocation of battery swapping stations and green charging facilities powered by renewable energy sources, including wind turbines and photovoltaic systems, to meet EV load demand. Unlike previous studies focusing solely on charging stations or isolated renewable energy sources, this study integrates both elements using a data clustering approach to enhance system performance. Additionally, the paper employs the PSO algorithm to optimize the placement and sizing of these facilities, aiming to maximize profitability while ensuring operational efficiency. Simulation results validate the effectiveness of the approach in achieving these objectives. Table 1 highlights the key attributes of previous studies alongside those of the present study, ensuring clarity at a glance and facilitating a comprehensive comparison.

**Table 1:** Comparison of the previous works.

References	Optimization objective	Type of optimization model	Method	Multiple station	Combined PV-Wind-BSwap	Distributed PV-Wind-BSwap	Green charging	Multi-criteria evaluation
^19^	Min cost	Linear programming (Location Allocation)	Queueing Model	✓	✗	✗	✗	✗
^23^	Max the total net revenue	Mixed-integer linear programming	Analytical model	✗	✓	✗	✓	✗
^24^	Min charging cost, waiting time, and CO₂ emissions	Metaheuristic	Multi-objective optimization	✓	✗	✗	✗	✗
^25^	Min costs	Mixed-integer linear programming	MILP-based algorithm	✓	✗	✗	✗	✗
^26^	Min operational costs	Mixed-integer linear programming	Real-time routing optimization	✓	✗	✗	✗	✓
^27^	Loss minimization and reliability enhancement	Mixed-integer nonlinear programming	Genetic algorithm	✓	✗	✗	✗	✓
^28^	Minimize the total operating cost	Two-stage stochastic programming	Stochastic optimization	✗	✗	✗	✗	✗
This study	Max net profit	Metaheuristic (optimal co-allocation)	Clustering-based optimization	✓	✓	✓	✓	✓

**Fig. 1:**
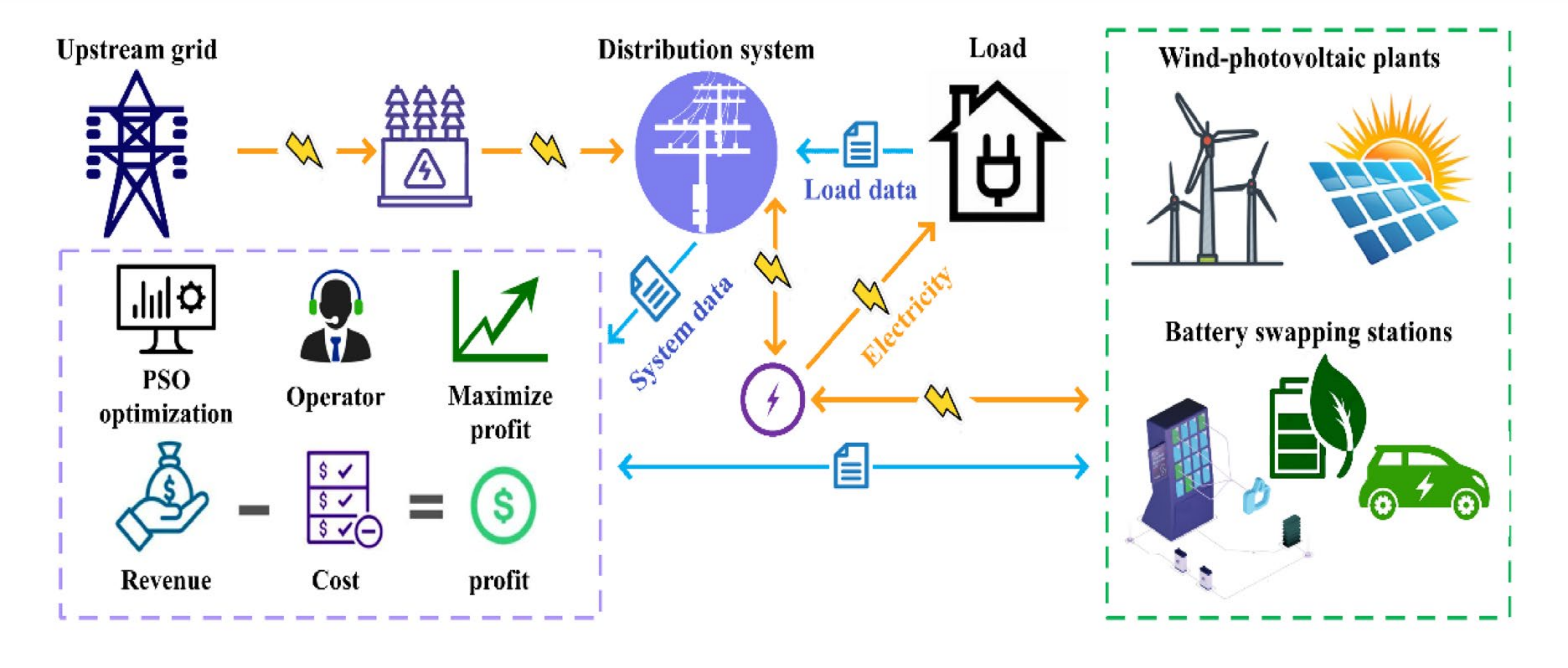
The illustrative schematic of the understudied problem.

The overall schematic of the problem under study is presented in Fig. 1. The primary contributions and novel aspects of this work are subsequently summarized as follows:

- Proposing an effective approach for the simultaneous co-allocation of battery swapping stations and wind-photovoltaic systems within radial distribution networks
- Employing the K-means algorithm and the elbow method to cluster the price, energy demand, wind, and photovoltaic generation optimally
- Maximizing net profit by balancing investment costs, loss costs, and revenue from energy sales, ensuring economic feasibility by utilizing the Particle Swarm Optimization (PSO) method
- Implementing the TOPSIS method for multi-criteria evaluation of the results based on key criteria, including minimum voltage, profit, energy loss, and renewable energy contribution

The structure of this paper is organized as follows: "System model" section presents a detailed description of the system model. Section 3 details the formulation of the proposed methodology. "Results" section discusses the simulation results, and "Conclusion" section concludes the study.

## System model

**Fig. 2:**
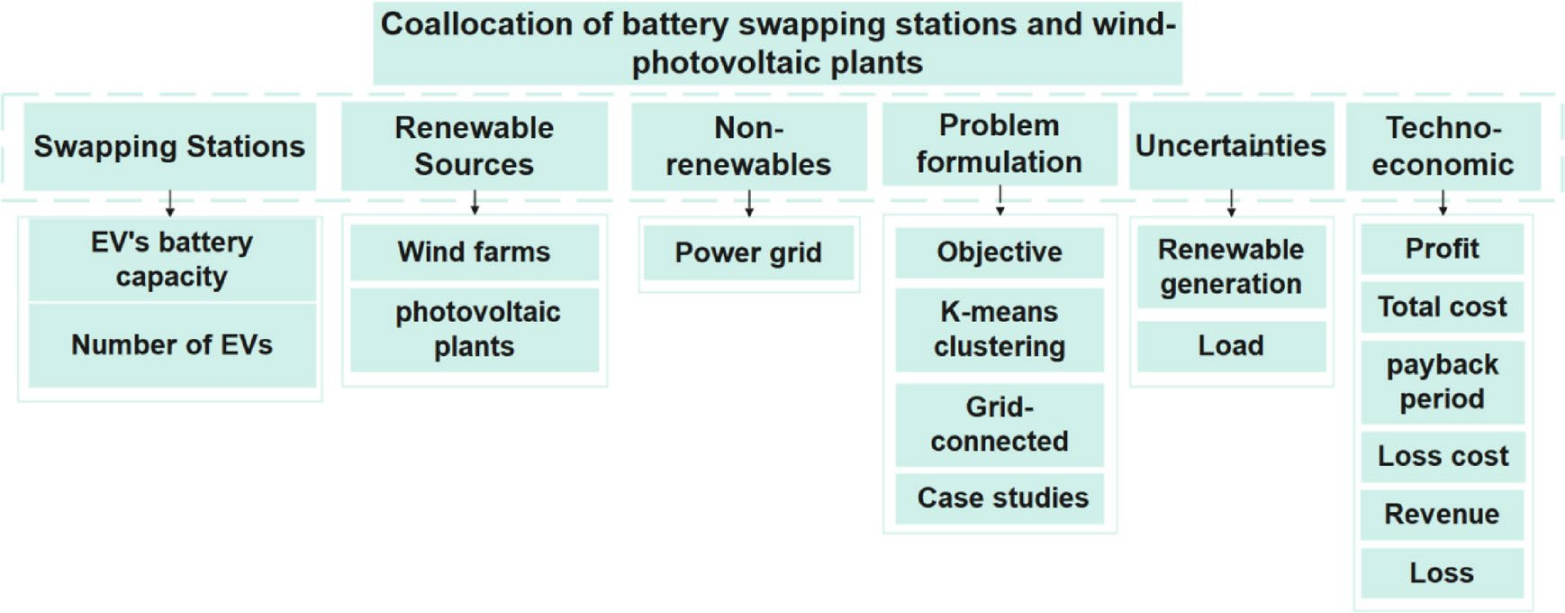
Highlights of the major focus of this study in the context of swapping stations integration utilization in distribution systems.

Figure 2 outlines the main focus of this study, which makes its comprehensiveness obvious. This work explores integrating swapping stations with different energy sources in distribution systems.

### Wind turbine model

Vertical-axis wind turbines are gaining popularity in urban areas due to their ability to harness power from multi-directional winds and their superior performance in turbulent conditions compared to horizontal-axis turbines. This paper focuses on vertical-axis wind turbines under 10 kW, valued for their affordability, low noise levels, and minimal infrasound emissions^29^.

**Fig. 3:**
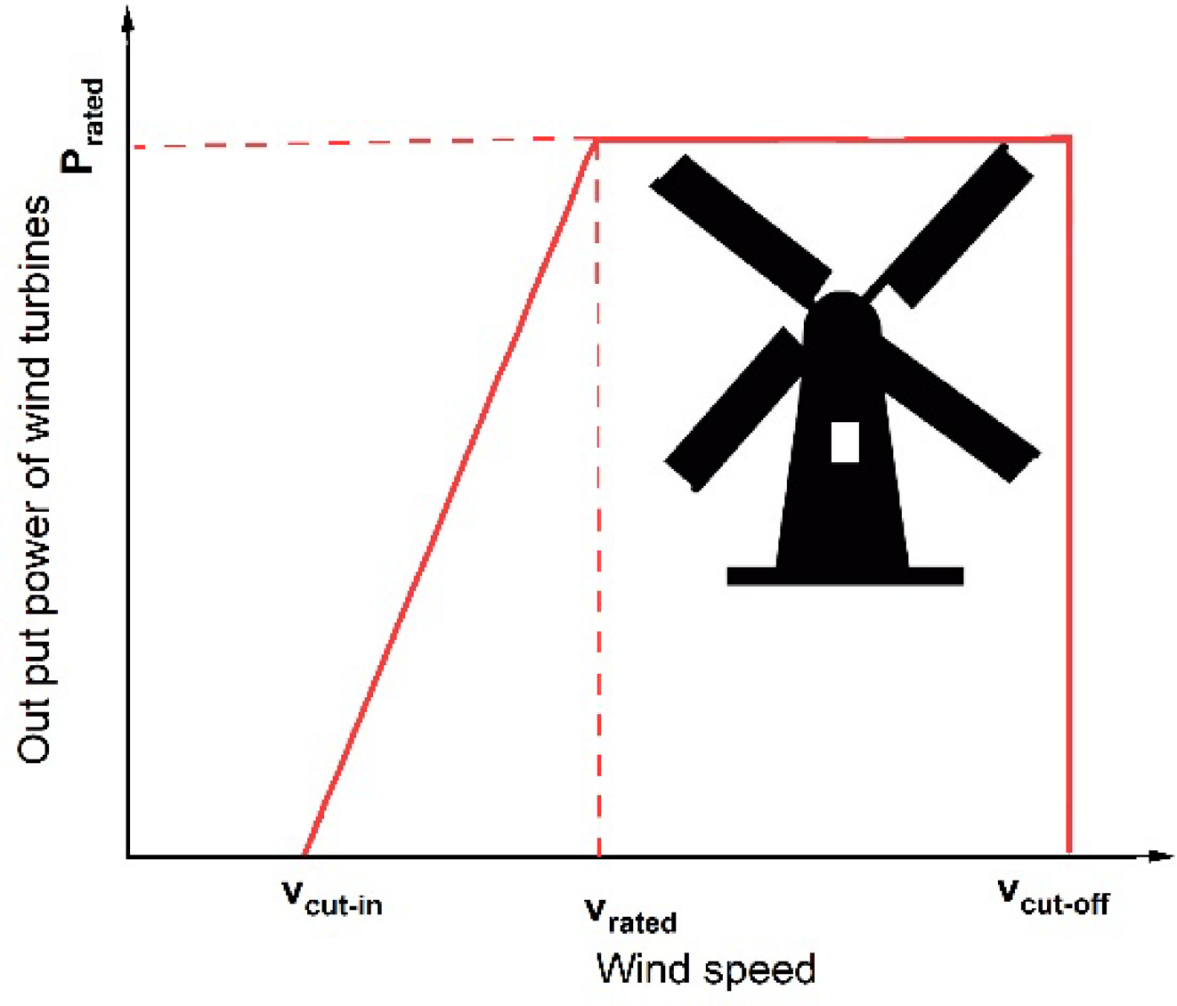
Wind power curve.

Figure 3 illustrates the variation of wind turbine output power with respect to wind speed. This relationship is mathematically expressed in Eq. (1):

1 $$P_{w} = \left\{ \begin{gathered} 0\,\,\,\,\,\,\,\,\,\,\,\,\,\,\,\,\,\,\,\,\,\,\,\,\,\,\,\,\,v \le v_{in} \,or\,v> v_{out} \hfill \\ x_{w} \frac{{v - v_{in} }}{{v_{r} - v_{in} }}\,\,\,\,\,\,\,\,v_{in} \le \,v \le v_{r} \hfill \\ P_{r} \,\,\,\,\,\,\,\,\,\,\,\,\,\,\,\,\,\,\,\,\,\,\,\,\,\,\,\,v_{r} \le v \le v_{out} \hfill \\ \end{gathered} \right.$$

The terms $v$, ${v}_{in}$, ${v}_{out}$, ${v}_{r}$, and ${x}_{w}$ represent the actual wind speed, cut-in wind speed, cut-out wind speed, nominal wind speed, and the wind turbine rated power, respectively. Furthermore, ${P}_{w}$ denotes the active output power of the wind turbine. The total output power of a wind farm, considering all wind turbines are operational, is the sum of the output power of each turbine. Therefore, the output power of the wind farm, ${P}_{w}^{F}$, for different configurations of wind turbines, can be expressed as:

2 $$P_{w}^{F} = \left( {N - N_{uw} } \right)P_{w}$$

**Fig. 4:**
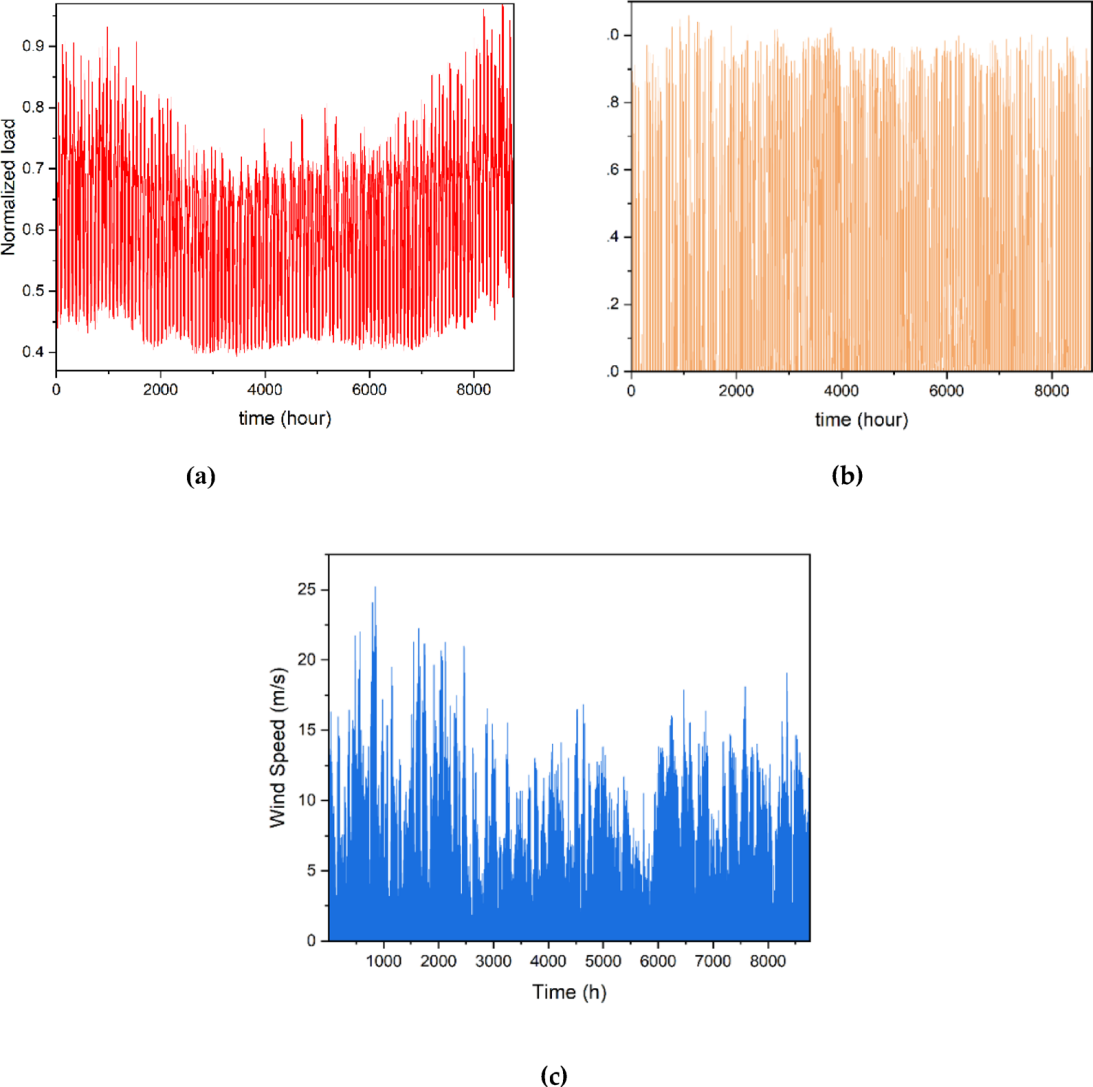
Hourly data for 1 year. (**a**) normalized load^21^, (**b**) solar irradiance, and (**c**) wind velocity^30^.

In this context, ${N}_{uw}$ and $N$ represent the number of wind turbines unavailable and the total number of wind turbines on the wind farm. Figure 4 illustrates the hourly load, corresponding hourly wind speed, and solar radiation levels. The data is compiled for an entire year, covering 8760 h.

### Solar cell model

Photovoltaic technology enables the direct conversion of sunlight into electricity and is the most commonly used method for generating power from solar radiation. Since 2002, the use of this technology has grown significantly, with an annual increase of 48%. Considering the influence of temperature on solar cell performance, it is essential to incorporate temperature variations into the analysis to accurately evaluate their behavior. The Nominal Operating Cell Temperature (NOCT) index addresses this^31^. NOCT represents the temperature of a solar cell under specific conditions: an environmental temperature of 20 °C, solar radiation of 0.8 kW/m^2^, and a wind speed of 4 m/s. The cell temperature is calculated using the following equation:

3 $$T_{PV} = T_{env} + \frac{NOCT - 20}{{0.8}}.\,S_{rad}$$

The temperature of the solar cell, denoted as ${T}_{PV}$, is measured in degrees Celsius and can be calculated using the ambient temperature ${T}_{env}$ and solar radiation ${S}_{rad}$, where max ${S}_{rad}$=1 kW/m^2^ under standard sunlight conditions. The output power of the solar cell is expressed using the following equation:

4 $$P_{PV} = P \times \left[ {\eta \times \left( {T_{PV} - 25} \right)} \right]$$

The efficiency of the solar cell in converting solar energy into electricity is represented by $\eta$. Figure 4b illustrates the solar radiation profile used in this study.

## The formulation and solution

### Load flow

The net active load at the bus interfaced with the battery swapping station and wind-photovoltaic systems (${P}_{{S}_{i}}$) within a ${C}_{bus}$-bus distribution network is computed by incorporating the stochastic generation from the wind farm (${P}_{{W}_{l}}^{F}$), photovoltaic system (${P}_{{PV}_{l}}^{F}$), the active power demand (${P}_{{d}_{i}}$), and the battery swapping station (${P}_{{BSwap}_{l}}$) corresponding to that bus. The relationship is expressed as follows:

5 $$P_{{s_{i} }} = \left\{ \begin{gathered} P_{{d_{i} }} \,\,\,\,\,\,\,\,\,\,\,\,\,\,\,\,\,\,\,\,\,\,\,\,\,\,\,\,\,\,\,\,\,\,\,\,\,\,\,\,\,\,\,\,\,\,\,\,\,\,\,\,\,\,\,\,\,\,\,\,\,\,\,\,\,\,\,\,\,\,\,\,\,\,\,\forall i \notin C_{bus} \hfill \\ P_{{d_{i} }} - N_{W}^{F} \,P_{{W_{l} }}^{F} - \,N_{PV}^{F} P_{{PV_{l} }}^{F} \, + \,\,P_{{BSwap_{l} }} \,\,\,\,\,\,\,\,\,\,\,\forall i \in C_{bus} \, \hfill \\ \end{gathered} \right.$$

As shown in Eq. (5), wind and solar energy sources are directly included in the load flow analysis of the distribution system. In practice, this means the energy generated by these sources is first directed to meet the nearby demand of the battery swapping station. Since the generation is located close to the load, this approach helps reduce power losses in the network. When the local demand—i.e., the energy demand of the battery swapping station—is fully satisfied, and surplus generation remains available, the excess power is allocated to other loads connected to the same distribution feeder, ensuring optimal utilization of renewable resources within the network. This method ensures that renewable energy is used efficiently, without unnecessary curtailment. It also reduces the power drawn from upstream sources and enhances the energy autonomy of the battery swapping station, decreasing its dependency on the grid. This energy flow exemplifies the typical operational behavior of distributed energy resources in practical systems, where local demands are prioritized, and surplus capacity is utilized to support the broader grid when available. This is a common feature in grid-connected renewable systems, particularly where energy policies support such integration. Rather than curtailing unused energy, which would waste potential clean generation, the model assumes that excess power is fed into the external grid. This provides a supplementary advantage by generating an additional revenue stream, thereby strengthening the economic justification for increased investments in renewable energy capacity.

The net reactive load at bus *i*, denoted as ${Q}_{i}$, and the reactive power demand at bus *i*, denoted as ${Q}_{{d}_{i}}$. The equation is formulated as follows:

6 $$Q_{i} = Q_{{d_{i} }} \,\,\,\,\,\,\,\,\,\,\,\,\,\,\,\,\,\,\,\,\,i = 1,2,...,N_{i}$$

The backward-forward sweep technique is employed in this study to carry out load flow calculations. Data clustering, the process of classifying data into distinct clusters based on similarities or differences, has led to the development of various approaches. This study uses the K-means method to cluster price, energy demand, wind, and photovoltaic generation.

### K-means data clustering

The sequential implementation of the K-means algorithm for data clustering^32^ is detailed as follows:

1. The number of clusters, $k$, and their respective centroids are randomly initialized based on the observations.
2. Assign the remaining observations to the nearest cluster of centroids using the following equation:

In this equation, ${a}_{m}$ and ${a}_{h}$ represent the cluster of centroids $m$ and $h$, respectively. $j$ denotes the total number of data points, while ${d}_{j}$ refers to the $j$ th data point, and ${D}_{h}$ signifies the set of members in the $h$-th cluster.

7 $$d_{j} \in D_{h\,\,\,\,\,\,} if\,\,\left| {d_{j} - a_{h} } \right| < \left| {d_{j} - a_{m} } \right|\,\,\,\,\,\,\,\,m = 1,2,...,M\,\,\,\,\,\,\,\,\,j = 1,2,...,J$$

3. The cluster centroids are updated by recalculating them based on the number of members (${N}_{S}$) in the *m*-th cluster, as specified by the following formula:
  8 $$a_{m} = \frac{{\sum\nolimits_{{j \in D_{h} }}^{{}} {d_{j} } }}{{N_{S} }}\,\,\,\,\,\,\,\,\,\,\,\,\,\,m = 1,2,...,M\,\,$$

This formula allows for updating cluster centroids by averaging the data points assigned to each cluster, ensuring that the centroids accurately represent the members within the cluster.

4. Steps 2 and 3 should be repeated until the centroid change is less than a predefined threshold for the cluster agents. This iterative process ensures the clustering stabilizes and converges to an optimal solution.
5. Once convergence is achieved, the probability of an agent *m* (${\omega }_{m}$) can be calculated using the following formula:
  9 $$\varpi_{m} = \frac{{c_{m} }}{J}$$
  where ${c}_{m}$ denotes the total number of data points within the *m*-th cluster. The primary goal of the K-means algorithm is to minimize the aggregate of squared distances between the cluster centroids and the corresponding observations, which is mathematically represented as follows:
  10 $$\min \sum\limits_{m = 1}^{k} {\sum\limits_{j = 1}^{{c_{m} }} {\left( {\left| {x_{j} - a_{m} } \right|} \right)} }^{2}$$
  where $k$ denotes the number of cluster centroids. The objective function computes the Euclidean distance between each data point ${x}_{j}$ and its corresponding centroid ${a}_{m}$. This metric plays a pivotal role in determining the quality of clustering by assessing the grouping of data points within each cluster, thereby guiding the optimization procedure of the K-means algorithm. In this study, the K-means clustering algorithm is employed to classify price, energy demand, wind, and photovoltaic generation data, effectively reducing the computational complexity of the analysis. This methodology has been extensively applied in numerous studies within the literature, particularly in the domain of distributed energy systems^30^.

To implement the K-means algorithm, the optimal number of clusters must be determined. This study adopts the elbow method for this purpose, as described in^33^. The elbow method involves plotting inertia against the number of clusters, where inertia, a critical metric for evaluating clustering quality, is defined as the sum of the squared distances between data points and their respective cluster centers. The elbow point signifies a significant reduction in inertia, indicating diminishing returns in clustering performance as the number of clusters increases. Beyond this point, additional clusters yield minimal improvement.

**Fig. 5:**
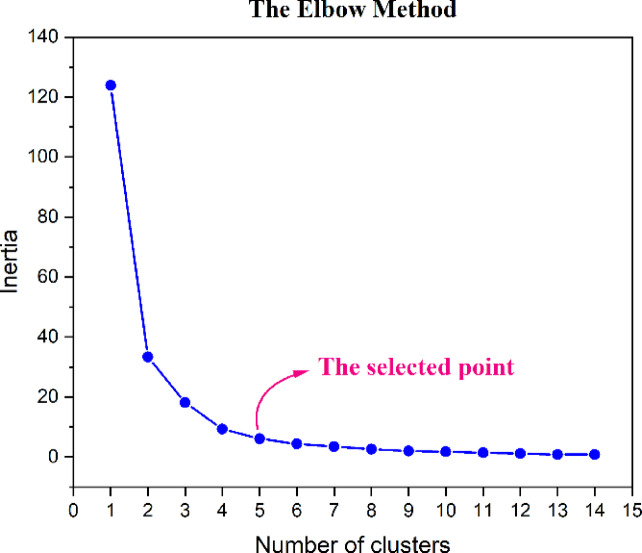
Determination of the optimal number of clusters using the elbow method in the K-means clustering algorithm.

Figure 5 depicts the relationship between inertia and the number of clusters in this analysis. Notably, between clusters 6 and 14, inertia demonstrates negligible variation. Consequently, point 5 is identified as the optimal choice, indicating that the K-means algorithm has classified the data into five clusters.

Given the significant temporal variability in the output power values of wind-photovoltaic systems, electricity prices, and load demand, the data are initially normalized to a range of (0, 1) using a single ratio derived from the maximum output values. Subsequently, the K-means algorithm is applied to classify the normalized data. The resulting cluster centroids are then scaled back to actual values by multiplying them by the maximum output vector, representing the peak values of wind and photovoltaic generation, followed by denormalization. Furthermore, the cluster centroids are weighted by their corresponding probabilities and the mean power output within each category, enabling the use of aggregated values as substitutes for individual data points.

### Optimization problem

The objective of the co-allocation problem involving swapping stations and wind-photovoltaic plants in radial distribution systems is to maximize profit (net profit). The investment costs associated with wind-photovoltaic plants and swapping stations depend on their per-unit size. Considering that the locations and capacities of swapping stations, wind farms, and photovoltaic systems are treated as discrete variables, the problem is structured as an integer optimization model. The mathematical formulation of the objective function is expressed as follows:

11 $$\max \;profit = revenue - cost$$

The revenue is expressed as:

12 $$\begin{aligned}revenue &= N_{T} \,.N_{y} \times \sum\limits_{m = 1}^{M}  \left( {\sum\limits_{i = 1}^{{N_{i} }} {\varpi_{m} \,} {\pi_{m} L_{{Grid_{i,m} }} } + \sum\limits_{i = 1}^{{N_{WF} }} {\pi_{m} \,.N_{W} .S_{W} .P_{{W_{i,m} }}^{{}} + } \sum\limits_{i = 1}^{{N_{SPP} }} {\,\pi_{m} \,.N_{PV} .S_{PV} .P_{{PV_{i,m} }}^{{}} + \pi_{m} \,.S_{BSwap} } } \right)  \\ L_{{Grid_{m} }} &=P_{{d_{m} }}-(P_{{W_{m} }}+P_{{PV_{m} }}-S_{BSwap})\end{aligned}$$

The size of the battery swapping station is defined as follows:

13 $$S_{BSwap} = N_{EV} \times S_{EV}$$

The total cost is outlined as:

14 $$C_{T} = C_{L} + C_{I}$$

The energy loss cost is formulated as follows:

15 $$C_{L} = \sum\limits_{m = 1}^{M} {\varpi_{m} \,} \pi_{m} .\,E_{Loss}$$

where ${\pi }_{m}$ represents the electricity price cluster, and ${E}_{Loss}$ denotes the total energy loss over the study period. The variable ${E}_{Loss}$ is calculated as follows:

16 $$E_{Loss} = N_{T} \,.N_{y} \times \sum\limits_{m = 1}^{M} {\varpi_{m} } \left( {\sum\limits_{i = 1}^{{}} {Z_{i + 1} \left| {I_{{L_{i + 1} }} } \right|^{2} } } \right)$$

Equation (17) represents the total investment cost, incorporating the expenditures associated with battery swapping stations and wind-photovoltaic systems.

17 $$C_{I} = C_{I,W} + C_{I,PV} + C_{I,BSwap}$$

The investment cost for the wind farms is defined as:

18 $$C_{I,W} = N_{W} \,S_{W} \,C_{SW}$$

The investment cost for the swapping stations is expressed as:

19 $$C_{I,BSwap\,} = N_{BSwap} \,S_{BSwap} \,C_{BSwap}$$

The investment cost for the photovoltaic plants is expressed as:

20 $$C_{I,PV} = N_{PV} \,S_{PV} \,C_{PV}$$

The limitations on swapping stations and wind-photovoltaic plants investment costs are outlined as follows:

21 $$C_{I,W} \le C_{{I,W_{\max } }}$$

22 $$C_{I,BSwap} \le C_{{I,BSwap_{\max } }}$$

23 $$C_{I,PV} \le C_{{I,PV_{\max } }}$$

The PSO algorithm is employed to solve the proposed optimization problem, with additional details provided in the Reference^34^. In the PSO methodology, the capacity of the wind-photovoltaic system and the placement of the hybrid system are defined as decision variables, with the objective function specified in Eq. (11). At each iteration, the optimization process identifies the most effective solution and selects the optimal bus for connecting swapping stations and wind-photovoltaic plants.

The proposed methodology for the optimal co-allocation of swapping stations and wind-photovoltaic plants consists of two main stages:

1. Data clustering: The first stage uses the K-means method to cluster the relevant data.
2. Optimization: In the final stage, the PSO algorithm is applied to optimize the formulated problem, thereby determining the optimal co-allocation of swapping stations and wind-photovoltaic plants in radial distribution systems.

**Fig. 6:**
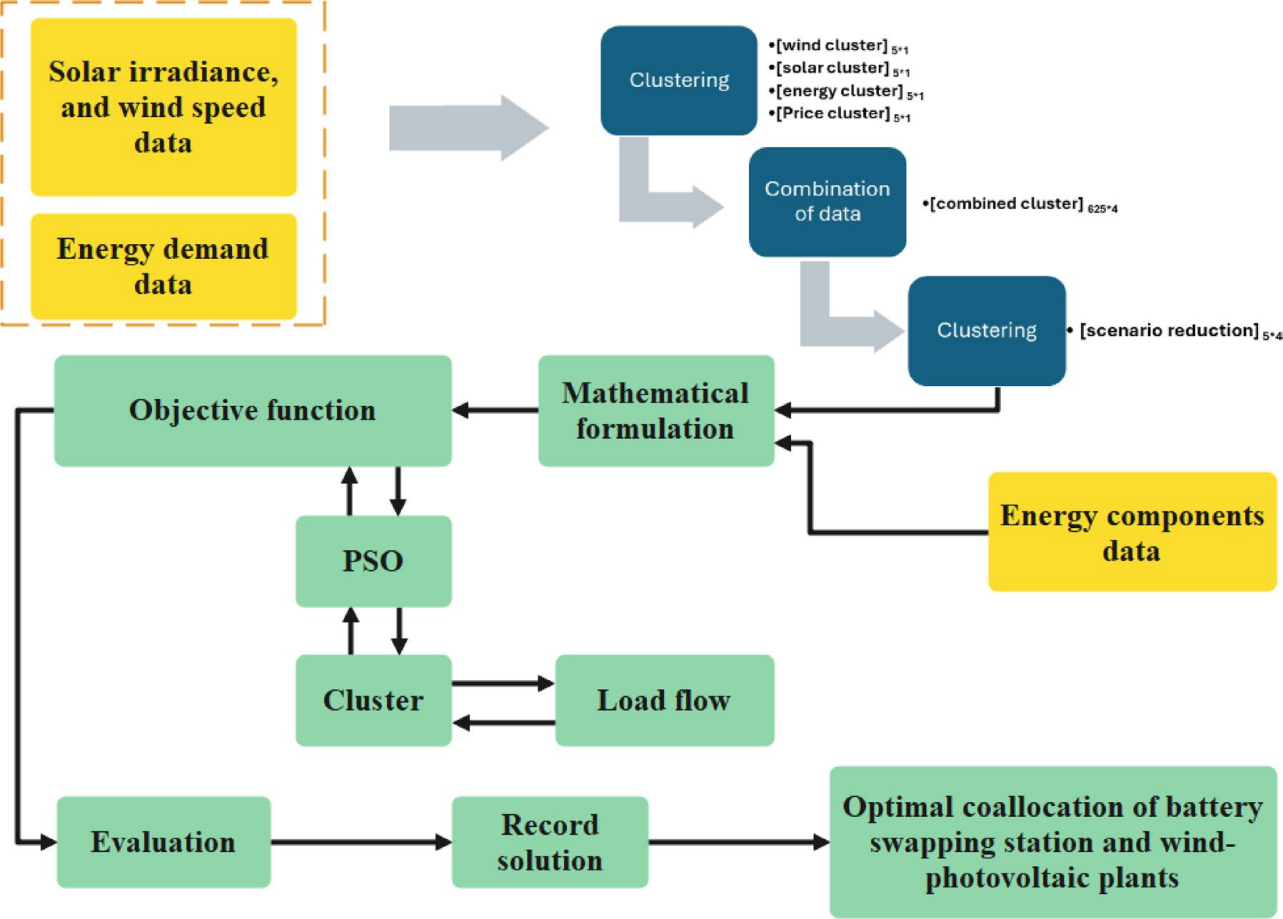
A framework for optimal co-allocation of swapping stations and wind-photovoltaic plants.

A framework, outlined in Fig. 6, presents the proposed methodology for addressing the co-allocation problem. The flowchart illustrates the input parameters of the optimization process, which include clustered data on energy demand, wind speed, and solar irradiance in the distribution system, along with the initial configurations of the PSO algorithm. The optimization process concludes once all PSO iterations are completed.

## Results

### Case studies

The proposed approach is evaluated by analyzing simulation results obtained from three distinct scenarios. Table 2 summarizes the scenarios examined in this study. The base-case scenario is employed to examine the distribution system’s behavior in the absence of swapping stations and wind-photovoltaic systems. In the first scenario, battery swapping stations are integrated into the network to assess their impact on operational parameters. The second scenario involves the optimal co-allocation of swapping stations and wind-photovoltaic systems within the radial distribution network. The presumptions and information needed to solve the optimization problem are described in this section. The outcomes are then reported.

1. The objective of the optimization problem is to identify the optimal placement of hybrid wind–photovoltaic–swapping station systems and to determine the optimal sizing of wind–photovoltaic systems.
2. Size of each battery swapping station was assumed to be 584 kW, enough to power 280 EVs (each with 25 kW power of the charger) over a 12-h day.
3. The IEEE 33-bus network is employed to assess the performance of the proposed methodology for the optimal co-allocation of wind-photovoltaic systems and battery swapping stations.

**Table 2:** State of the system in different scenarios.

Scenario	Explanation of the system under study
Base case	The IEEE 33-bus radial distribution network is configured without the integration of swapping stations and wind-photovoltaic systems
First scenario	The system with swapping stations
Second scenario	The system with optimal co-allocation of swapping stations and wind-photovoltaic plants

**Fig. 7:**
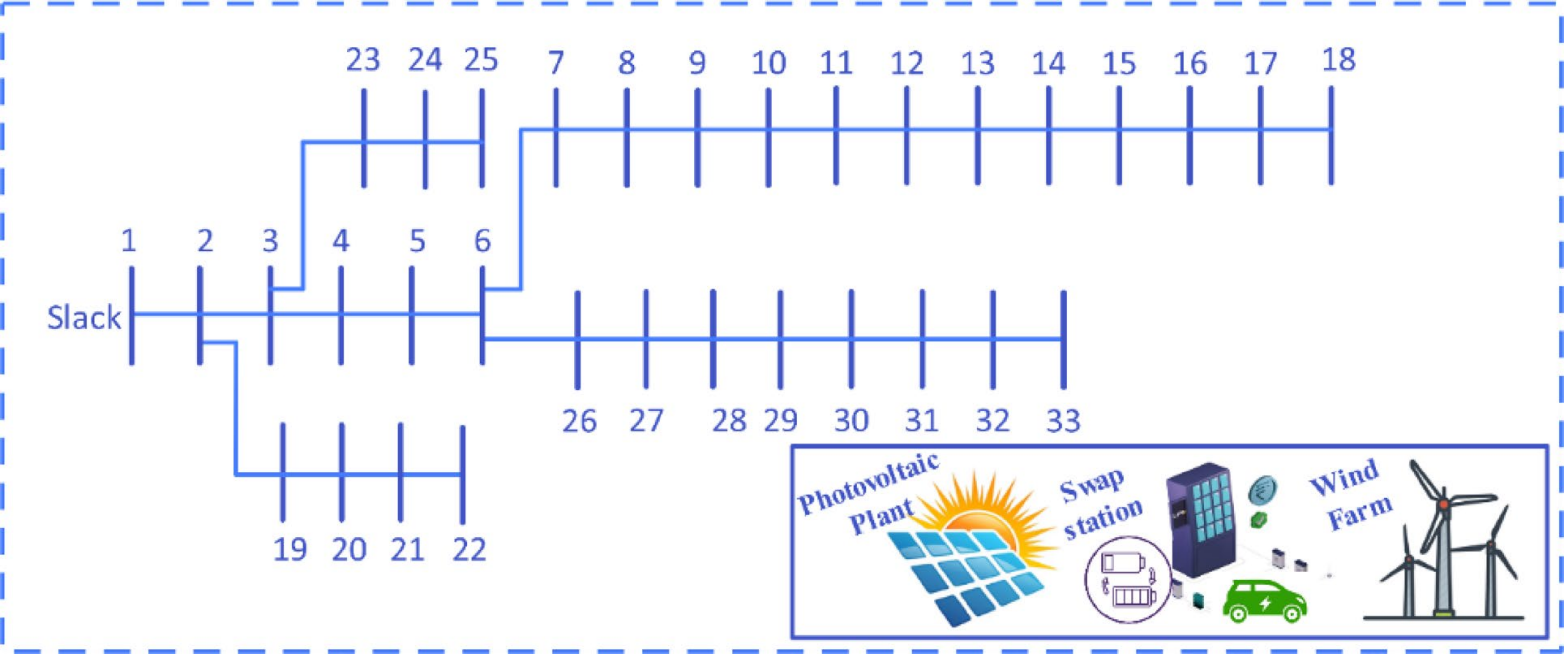
Single-line representation of the IEEE 33-bus radial distribution network.

Figure 7 depicts the single-line representation of the IEEE 33-bus radial distribution system. Table 3 outlines the cost factors associated with battery swapping stations and wind-photovoltaic systems required for optimal investment. The simulation studies were performed using MATLAB, executed on a system equipped with an Intel Core i7 processor operating at 2.60 GHz with 16 GB of RAM. The parameters pertaining to the wind turbine are detailed in Table 4.

**Table 3:** The cost factors for swapping station and wind-photovoltaic plant.

Cost factors of:	
Wind turbine	$${C}_{SW}$$: $$800$$ $/kW
Photovoltaic panel	$${C}_{PV}$$: $$800$$ $/kW
Battery swapping station	$${C}_{SS}$$: 5 $$00$$ $/kW

**Table 4:** Specifications of wind turbines.

Active output power	*P*_*r*_ = 10 kW
Cut-in wind speed	*v*_*in*_ = 4 m/s
Cut-out wind speed	*v*_*out*_ = 22 m/s
Nominal wind speed	*v*_*r*_ = 10 m/s

The comparison focuses on three key aspects: increased profit, improvement in voltage profiles, and the overall computational burden. It should be noted that the outputs of the proposed technique are specifically designed for planning applications. A clustering-based framework is employed in conjunction with the PSO algorithm to determine the optimal co-allocation of swapping stations and wind-photovoltaic plants. The decision variables for the PSO algorithm encompass both the capacity of the wind-photovoltaic system and the placement of the integrated hybrid system. The objective function is formulated to maximize the network profit, with the method’s adjustable parameters set as follows: number of generations = 100 and number of particles = 1000.

**Fig. 8:**
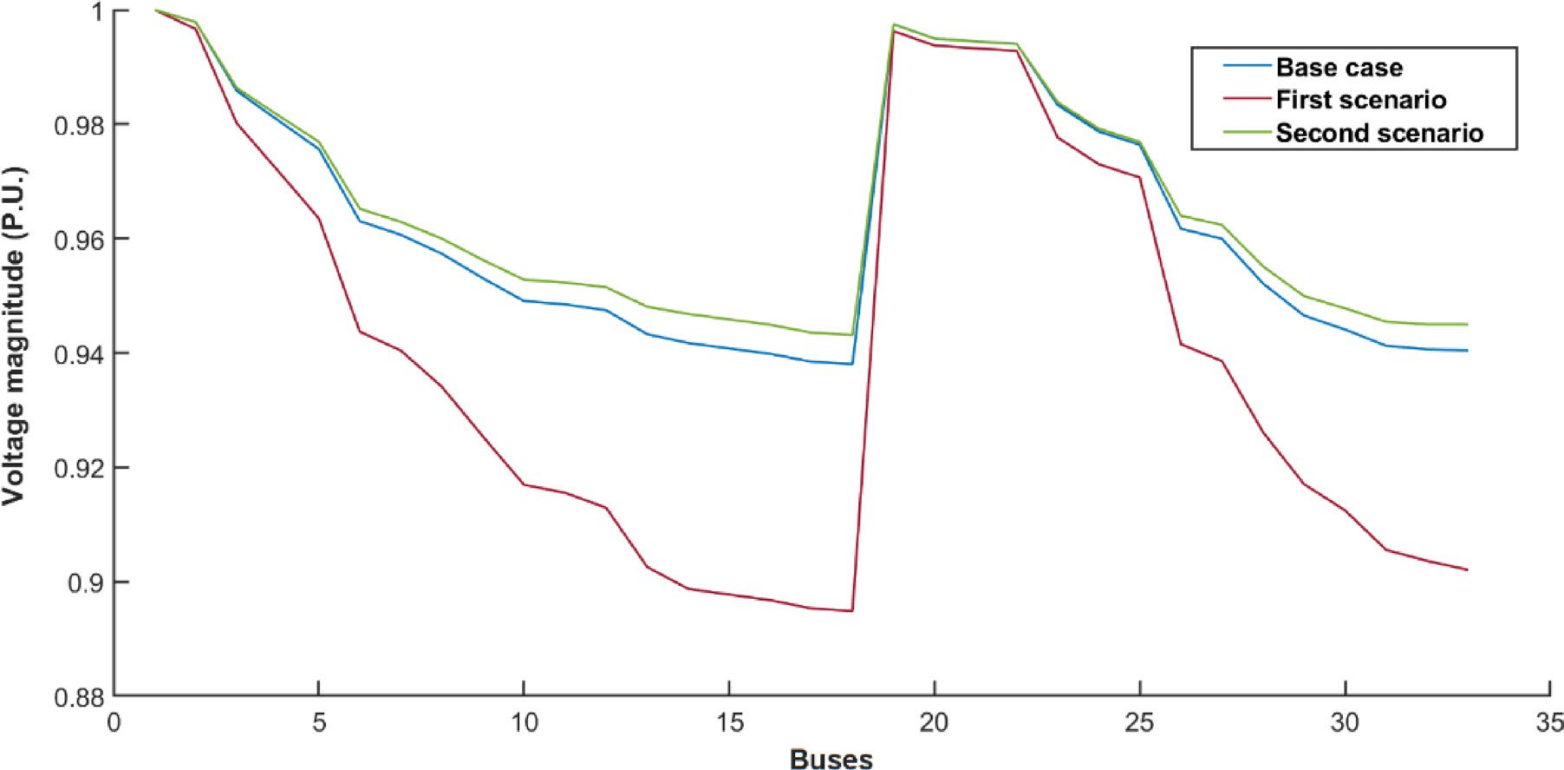
Voltage profile of the case studies.

The IEEE 33-bus radial distribution system is employed to further validate the effectiveness of the proposed methodology. For the IEEE 33-bus distribution system, as illustrated in Table 5, buses 2, 33, and 16 are identified as the optimal nodes for connecting the swapping stations and wind-photovoltaic plants. The optimal size of wind farms is 600, 1200, and 1100 kW. The optimal size of photovoltaic systems is 600, 900, and 1200 kW. Renewable energy sources are integrated into the grid, fulfilling a dual role: meeting the energy demands of battery swapping stations and partially supplying the distribution system. This reduces energy loss and reliance on the upstream grid. Table 5 additionally provides an estimation of the computational time associated with solving the proposed co-allocation problem. In summary, the data clustering methodology proves to be effective for the co-allocation of swapping stations and wind-photovoltaic plants, even when applied to large-scale test systems. Figure 8 depicts the voltage profile of the second scenario in the optimal state, facilitating a comparison with other scenarios. The baseline curve shown in this figure corresponds to the condition in which the swapping stations and wind-photovoltaic plants are not integrated into the network. As shown, the voltage profile improves across all buses in the network after installing the wind-photovoltaic plants compared to the first scenario.

**Table 5:** The optimal results for a 33-bus system over a ten-year period.

Scenario	Base case	First scenario	Second scenario
Energy loss (GWh)	8.4814	23.2333	7.4979
Loss cost (k$)	508.8827	1393.9995	449.8743
Investment (k$)	–	876	5354.995
Total cost (k$)	508.8827	2268.9945	5804.8693
Revenue (k$)	3748.9997	9635.686	15051.4684
Profit (k$)	3240.117	7366.6916	9246.5992
Total payback period (years)	–	0.91	3.56
Time burden (min)	–	–	24
Renewable share	–	–	22%
Optimal buses for wind-photovoltaic plants	–	–	2, 33, and 16
Optimal size of wind farms (kW)	–	–	600,1200, and 1100
Optimal size of photovoltaic plants (kW)	–	–	600,900, and 1200
Buses for swapping stations	–	2, 33, and 16	2, 33, and 16
Capacity of swapping stations (kWh)	–	584, 584, 584	584, 584, 584

### Sensitivity analysis

**Fig. 9:**
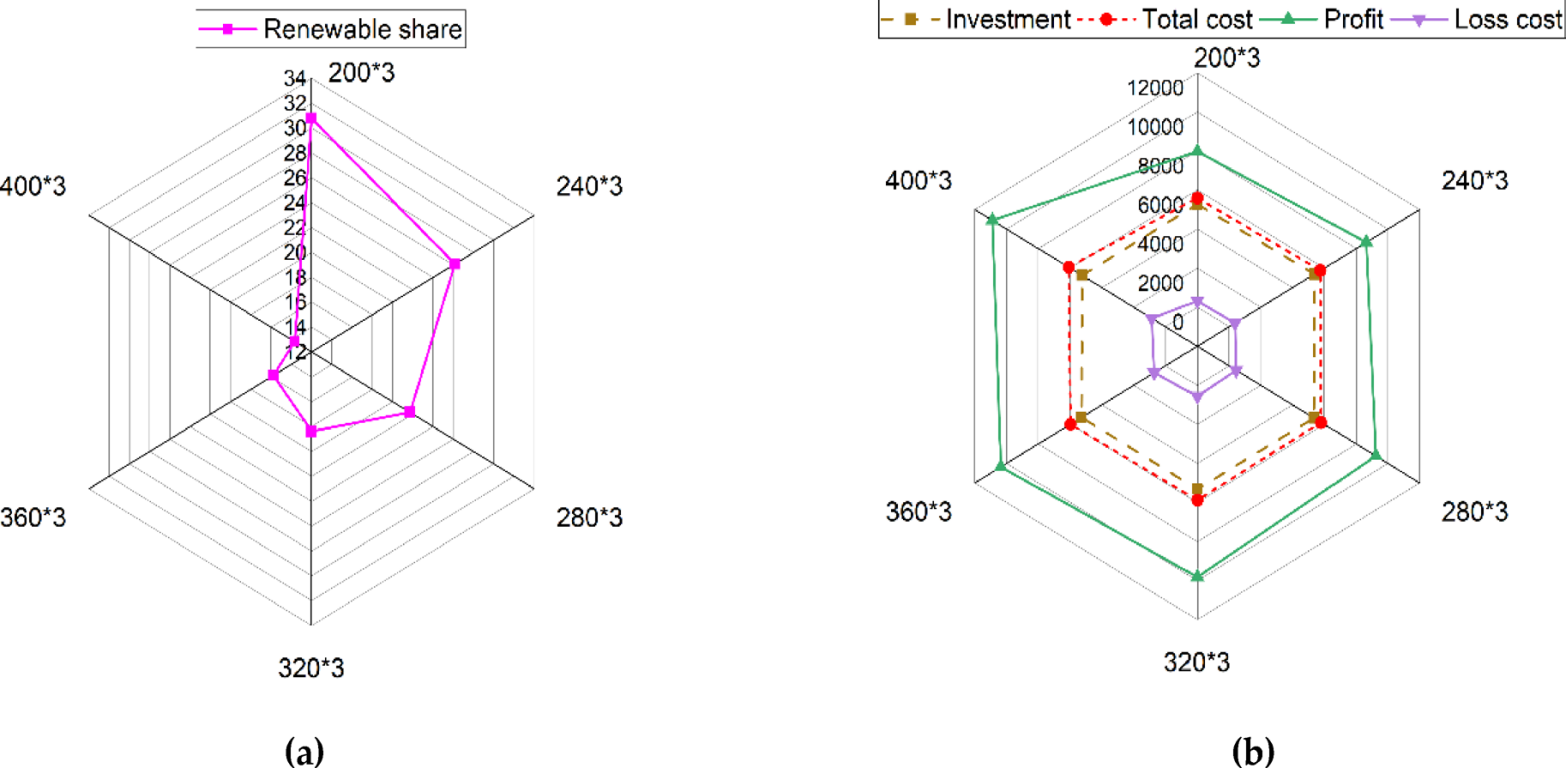
Effects of the number of EVs on the: (**a**) renewable share, and (**b**) profit, total cost, investment, and loss cost.

**Fig. 10:**
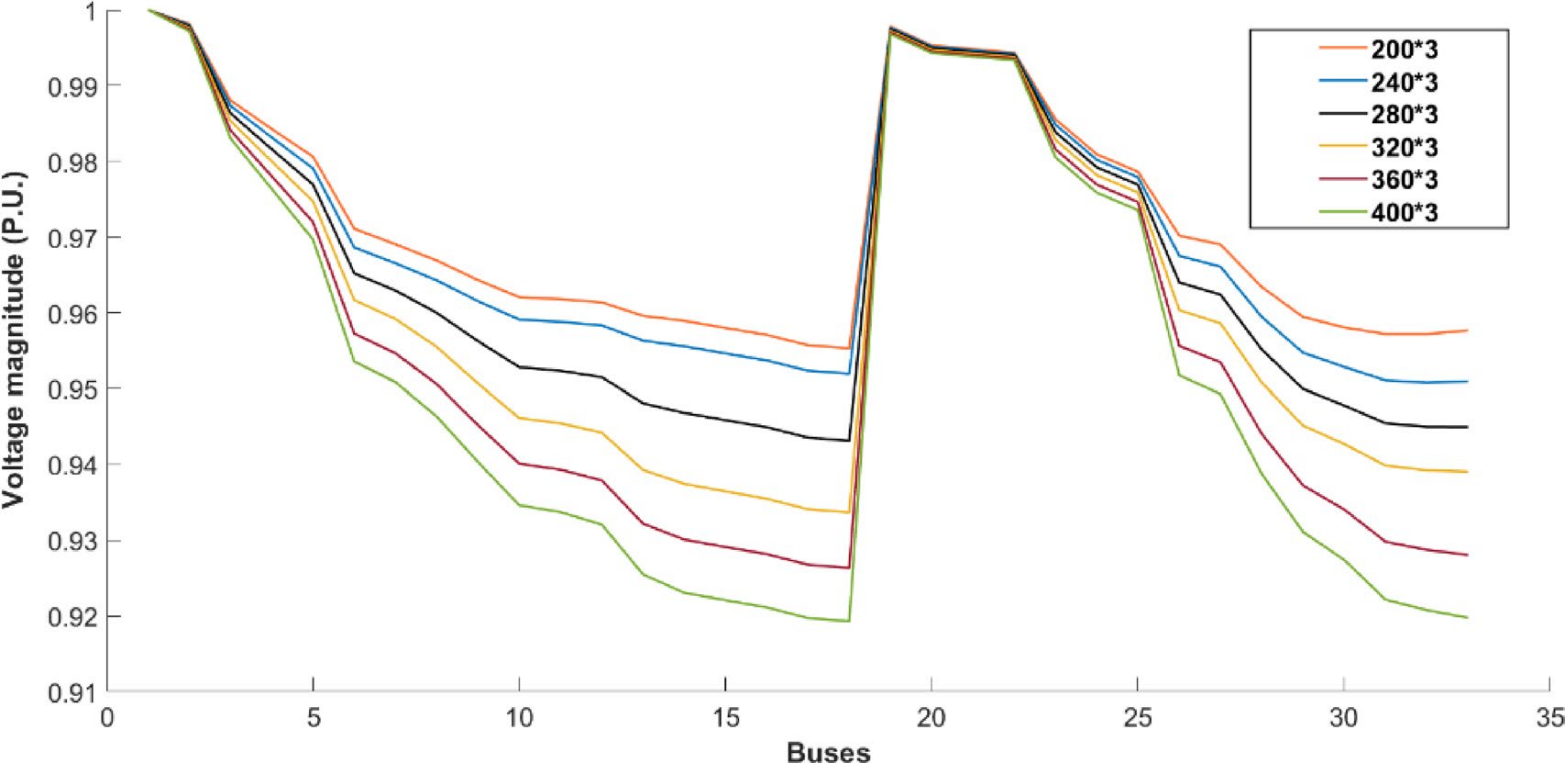
Voltage profile of the buses in various numbers of EVs with swapping stations and wind-photovoltaic plants.

A sensitivity analysis is conducted to assess the impact of the number of EVs on the optimization outcomes of the proposed co-allocation strategy for swapping stations and wind-photovoltaic plants. Figure 9a shows the effects of the number of EVs on the share of the wind-photovoltaic plants to supply the total energy demand, which changes between 30.78% and 13.67%. The size of the battery swapping station can significantly impact the system’s economic performance. Figure 9b illustrates the relationship between profit, total cost, investment, and loss cost across different numbers of EVs. The profit increases from 7973.07 to 10,889.79 k$ as the capacity of each battery swapping station grows from 417 kWh (sufficient for 200 EVs) to 834 kWh (sufficient for 400 EVs) with limited investment cost. This growth is achieved with a limited investment cost of 5400 k$. Figure 10 illustrates the impact of varying the number of electric vehicles (EVs) on the voltage profile within the framework of the proposed co-allocation strategy for swapping stations and wind-photovoltaic plants. The results demonstrate that an increase in the number of EVs leads to a corresponding decline in the voltage profile.

### TOPSIS ranking

The previous section discussed the impact of the number of EVs on the optimization results for the proposed co-allocation of swapping stations and wind-photovoltaic plants. The results indicate that increasing the number of EVs improves profitability while concurrently lowering the minimum voltage and diminishing the contribution of wind-photovoltaic plants in meeting the overall energy demand. A quantitative comparison of the various test cases is essential for evaluating the results while accounting for all critical parameters that influence system performance. These criteria include minimum voltage (Vmin), profit, loss, and energy contribution from wind-photovoltaic plants. The Technique for Order of Preference by Similarity to Ideal Solution (TOPSIS), a multi-criteria decision-making methodology, is utilized to assess and analyze the results based on the specified criteria^35^. The decision matrix $R=\left\{{r}_{ij}\left| i=\text{1,2},\dots ,m;j=\text{1,2},\dots ,n\right.\right\}$, comprising $m=6$ criteria and $n=5$ alternatives, is displayed in Table 6.

**Table 6:** TOPSIS decision matrix.

Number of EVs	V_min_ P.U	Criteria Profit k$	Loss kW	Renewable share %
$$N_{EV} = 200*3$$	0.955294	7973.0715	5.4146	30.786
$$N_{EV} = 240*3$$	0.950753	8642.9659	6.1833	26.1575
$$N_{EV} = 280*3$$	0.945129	9246.5992	7.4979	21.728
$$N_{EV} = 320*3$$	0.93366	9816.142	9.3807	18.3842
$$N_{EV} = 360*3$$	0.926327	10,356.7799	11.7467	15.7703
$$N_{EV} = 400*3$$	0.919273	10,889.7876	14.2384	13.6712

Following the construction of the weighted normalized decision matrix as defined by Eq. (24), the maximum ($b^{ + }$) and minimum ($b^{ - }$) values for each criterion are identified.

24 $${\text{B}} = \left( {b_{ij} } \right)_{m \times n} = \frac{{r_{ij } . wf_{j} }}{{\sqrt {\mathop \sum \nolimits_{i = 1}^{n} r_{ij}^{2} } }}$$

**Fig. 11:**
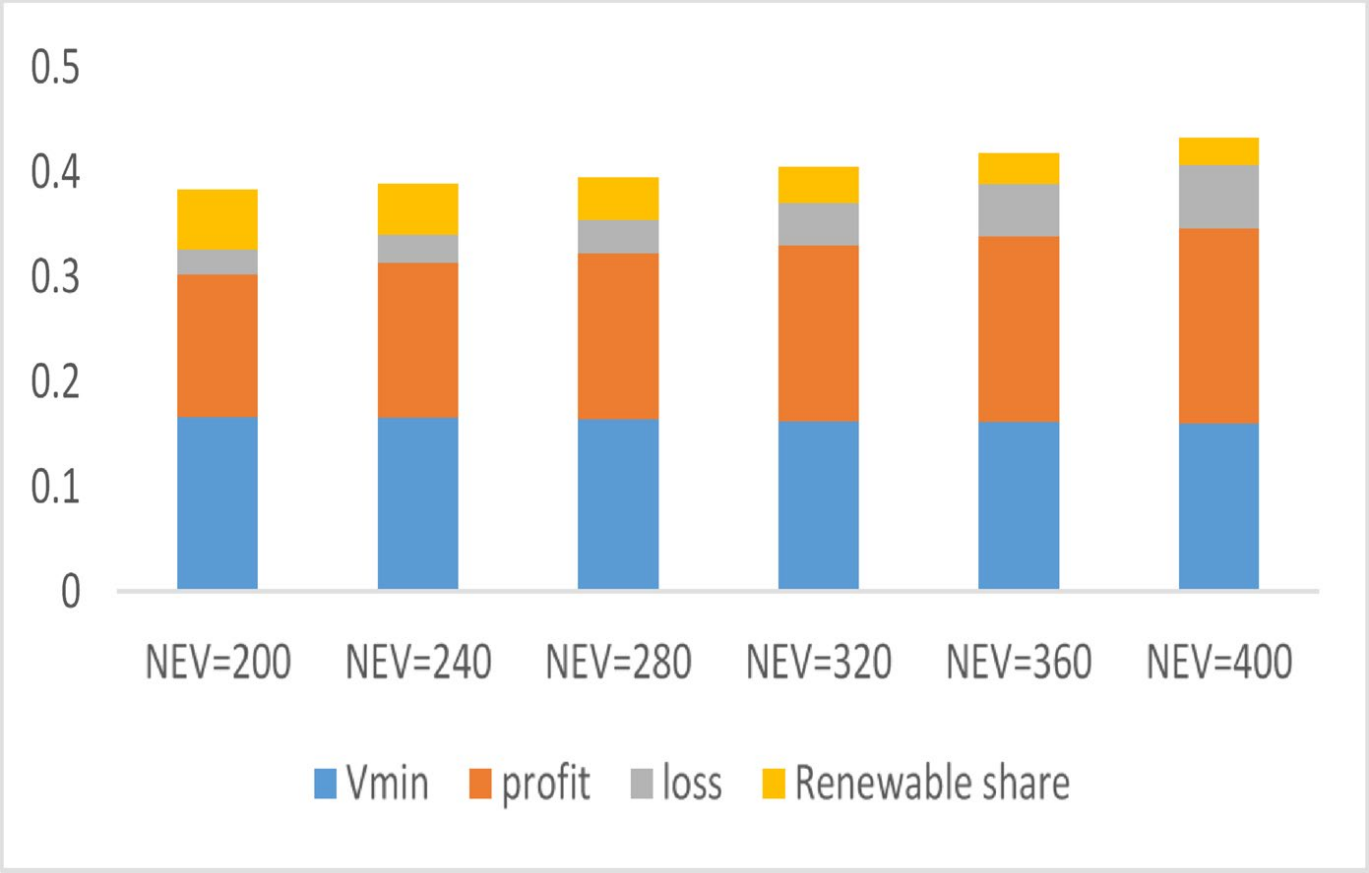
The score of oscillators on each criterion.

The weighted normalized decision matrix is shown in Table 7, along with the weight values assigned to the selected criteria. Since each criterion reflects a distinct aspect of the oscillators’ performance, assigning equal weights to all criteria would not accurately represent their importance. Table 7 presents the weight factors, highlighting the greater significance of profit and minimum voltage (Vmin) in this study, as recommended by previous research^36^. The weights assigned to profit and minimum voltage are set at 0.4, reflecting their greater importance compared to loss and renewable share, which are both weighted at 0.1. Figure 11 illustrates the scores of oscillators for each criterion, as detailed in Table 7.

**Table 7:** The weighted normalized decision matrix.

Criteria	V_min_ P.U	Profit k$	Loss kW	Renewable share %
Number of EVs	Weight			
	0.4	0.4	0.1	0.1
$$N_{EV} = 200*3$$	0.166282	0.136491	0.023032	0.057389
$$N_{EV} = 240*3$$	0.165491	0.147959	0.026302	0.048761
$$N_{EV} = 280*3$$	0.164164	0.158279	0.031948	0.040504
$$N_{EV} = 320*3$$	0.162516	0.168042	0.039903	0.034271
$$N_{EV} = 360*3$$	0.161239	0.177298	0.049967	0.029398
$$N_{EV} = 400*3$$	0.160012	0.186422	0.060566	0.025485

oscillators listed in Table 7, the Euclidean distances between each matrix element and the optimal and suboptimal solutions are calculated in compliance with Eqs. (25) and (26).

25 $$h_{i}^{ + } = \left[ {\sum\limits_{j = 1}^{m} {\left( {b_{ij} - b_{j}^{ + } } \right)^{2} } } \right]^{0.5}$$

26 $$h_{i}^{ - } = \left[ {\sum\limits_{j = 1}^{m} {\left( {b_{ij} - b_{j}^{ - } } \right)^{2} } } \right]^{0.5}$$

The oscillators are prioritized according to their proximity to the worst solution, as defined by Eq. (27). Table 7 demonstrates that higher values correspond to oscillators achieving conditions closer to the optimal state.

27 $$S_{i} = \frac{{h_{i}^{ - } }}{{h_{i}^{ + } + h_{i}^{ - } }}$$

**Fig. 12:**
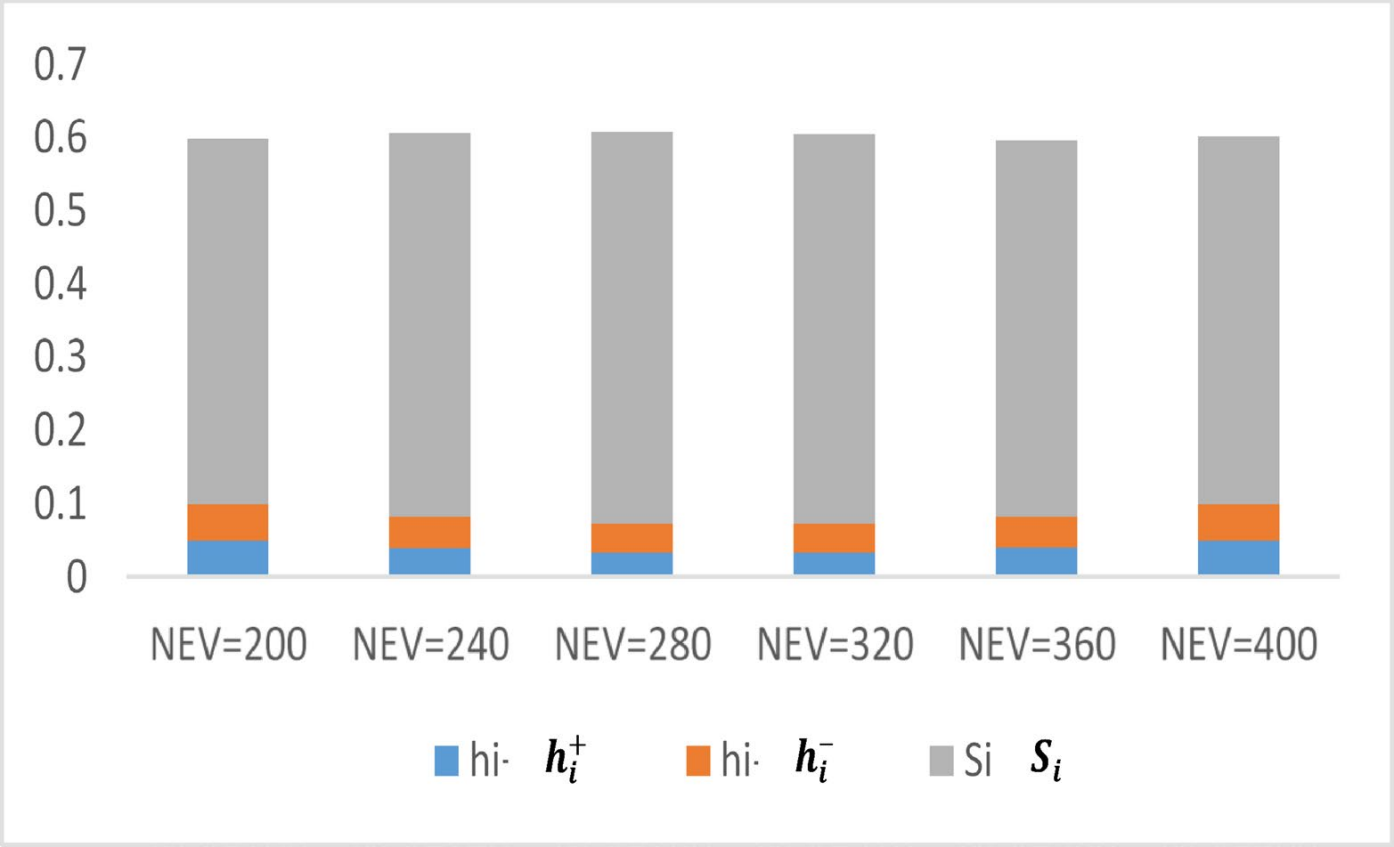
Closer proximity of oscillators to the optimal condition.

The Euclidean distances of each alternative to the optimal solution (${h}_{i}^{+}$) and the least favorable solution (${h}_{i}^{-}$) are calculated to assess their proximity to the ideal solution (${S}_{i}$). The analysis enables the ranking of various test items based on their proximity to the optimal solution. The values presented in Table 8 offer critical insights into the comparative performance of multiple oscillators. Figure 12 illustrates the proximity of numerous oscillators to the optimal condition. As shown, the oscillator corresponding to 280 EVs achieves the highest rank.

**Table 8:** The Euclidean distances of each alternative to the optimal and least favorable solutions, as well as their proximity to the ideal solution, are employed to establish the rankings of various test items.

Number of EVs	$h_{i}^{ + }$	$h_{i}^{ - }$	$S_{i}$	Rank
$N_{EV} = 200*3$	0.049931	0.049494	0.5	6
$N_{EV} = 240*3$	0.039574	0.043176	0.52	3
$N_{EV} = 280*3$	0.034056	0.039103	0.55	1
$N_{EV} = 320*3$	0.034135	0.038775	0.53	2
$N_{EV} = 360*3$	0.040083	0.04236	0.51	4
$N_{EV} = 400*3$	0.049485	0.049943	0.5	5

## Conclusion

This study presents a data clustering-based methodology aimed at achieving the optimal co-allocation of battery swapping stations and wind-photovoltaic systems within radial distribution networks. The K-means method clusters price, energy demand, wind, and photovoltaic generation. Implementing the K-means algorithm requires determining the optimal number of clusters. This study uses the elbow method for this purpose. The proposed methodology employs data clustering techniques to enhance execution efficiency and minimize computational expenses. The main objective is to maximize net profit through the optimization process facilitated by the PSO algorithm. The effectiveness of the proposed approach is evaluated on the standard IEEE 33-bus test system, considering the base case and two distinct operational conditions. A quantitative comparison of various test cases is required to evaluate the results by considering all key influence criteria on the system’s performance. This study employs the TOPSIS method to assess the results based on key criteria, including minimum voltage, profit, energy loss, and renewable energy contribution.

## Data Availability

All data generated or analyzed during this study are included in this published article.

## List of symbols

$$i$$: Bus index
$c_{m}$: Total member count in cluster *m*
$C_{SW}$: Cost per unit size of the wind turbine
$S_{W}$: Size of wind turbines
$N_{W}$: Total number of wind turbines
$P_{{W_{i,m} }}^{{}}$: Wind farm generation cluster
$N_{WF}$: Number of wind farms
$S_{BSwap}$: Battery swapping station size
$C_{BSwap}$: Cost per unit size of the battery swapping station
$N_{PV}$: Total number of solar panels
$S_{PV}$: Size of photovoltaic panels
$P_{{PV_{i,m} }}^{{}}$: Photovoltaic system generation cluster
$N_{SPP}$: Number of photovoltaic plants
$l_{f1} ,l_{f2}$: Learning factors of PSO
$D_{h}$: Set of members in cluster *h*
$$d_{j}$$: Data point *j*
$$m,\,h$$: Clusters index
$I_{{L_{i} }}$: The current of line *i*
$$C_{I}$$: Investment cost
$C_{I,W}$: The investment cost for wind farms
$C_{{I,W_{\max } }}$: Maximum investment for wind farms
$C_{I,BSwap}$: The investment cost for the battery swapping station
$C_{{I,BSwap_{\max } }}$: Maximum investment for battery swapping station
$C_{I\,,PV}$: The investment cost for photovoltaic plants
$C_{{I,PV_{\max } }}$: Maximum investment for photovoltaic plants
$j$: Data points index
$M$: Total count of clusters
$L$: The total loss over the analyzed time horizon
$C_{L}$: Cost of loss
$J$: The total count of data points
$N_{S}$: The total count of members
$P_{i}$: The active load at bus* i*
$\pi_{m}$: Electricity price cluster
$P_{W}^{F}$: The output power of wind farm
$P_{W}$: The output power of wind turbine
$L_{{Grid_{i,m} }}$: Power purchase from the main grid
$\varpi_{m}$: Probability of agent *m*
$$N_{T}$$: Planning period
$Q_{i}$: Reactive load of bus *i*
$x_{w}$: Size of wind turbines
$T_{y}$: The cumulative duration in years
$$C_{T}$$: Total cost
$V_{i}$: The voltage of bus *i*
$v_{in}$: The wind turbine’s cut-in wind speed
$v_{out}$: The wind turbine’s cut-out wind speed
$v_{r}$: The wind turbine’s nominal wind speed
$Z_{i}$: Impedance of line *i*

## Abbeviations

PSO: Particle swarm optimization
EV: Electric vehicle
